# Recreational water exposure and waterborne infections in a prospective salivary antibody study at a Lake Michigan beach

**DOI:** 10.1038/s41598-021-00059-2

**Published:** 2021-10-15

**Authors:** Andrey I. Egorov, Reagan Converse, Shannon M. Griffin, Russell Bonasso, Lindsay Wickersham, Elizabeth Klein, Jason Kobylanski, Rebecca Ritter, Jennifer N. Styles, Honorine Ward, Elizabeth Sams, Edward Hudgens, Alfred Dufour, Timothy J. Wade

**Affiliations:** 1 United States Environmental Protection Agency, Chapel Hill, NC USA; 2 United States Environmental Protection Agency, Cincinnati, OH USA; 3 University of North Carolina at Chapel Hill, Chapel Hill, NC USA; 4 Tufts Medical Center, Boston, MA USA

**Keywords:** Infection, Epidemiology

## Abstract

In a prospective observational study, seroconversion to a specific pathogen can serve as a marker of an incident infection, whether or not that infection is symptomatic or clinically diagnosed. While self-reported symptoms can be affected by reporting bias, seroconversion is likely to be free of this bias as it is based on objective measurements of antibody response. Non-invasive salivary antibody tests can be used instead of serum tests to detect seroconversions in prospective studies. In the present study, individuals and families were recruited at a Lake Michigan beach in Wisconsin in August 2011. Data on recreational water exposure and baseline saliva samples (S1) were collected at recruitment. Follow-up data on gastrointestinal symptoms were collected via a telephone interview approximately 10 days post-recruitment. Follow-up saliva samples were self-collected approximately 2 weeks (S2) and 30–40 days post-recruitment (S3) and mailed to the study laboratory. Samples were analyzed for immunoglobulin (Ig) G responses to recombinant antigens of three noroviruses and *Cryptosporidium*, as well as protein purification tags as internal controls, using an in-house multiplex suspension immunoassay on the Luminex platform. Responses were defined as ratios of antibody reactivities with a target protein and its purification tag. Seroconversions were defined as at least four-fold and three-fold increases in responses in S2 and S3 samples compared to S1, respectively. In addition, an S2 response had to be above the upper 90% one-sided prediction limit of a corresponding spline function of age. Among 872 study participants, there were seven (0.8%) individuals with seroconversions, including six individuals with seroconversions to noroviruses and two to *Cryptosporidium* (one individual seroconverted to both pathogens). Among 176 (20%) individuals who reported swallowing lake water, there were six (3.4%) seroconversions compared to one (0.14%) seroconversion among the remaining 696 individuals: the crude and age-standardized risk differences per 1000 beachgoers were 32.7 (95% confidence limits 5.7; 59.6) and 94.8 (4.6; 276), respectively. The age-adjusted odds ratio of seroconversion in those who swallowed water vs. all others was 49.5 (4.5; 549), p = 0.001. Individuals with a norovirus seroconversion were more likely to experience vomiting symptoms within 4 days of the index beach visit than non-converters with an odds ratio of 34 (3.4, 350), p = 0.003. This study contributed further evidence that recreational water exposure is associated with symptomatic and asymptomatic waterborne infections, and that salivary antibody assays can be used in epidemiological surveys of norovirus and *Cryptosporidium* infections.

## Introduction

Swimming in recreational surface water bodies in the US has been linked with outbreaks of infectious gastroenteritis^1,2^. Many of these outbreaks were caused by noroviruses and *Cryptosporidium* spp. These pathogens are present in sewage at high concentrations and they persist in surface water^3,4^. Epidemiological studies have also linked recreational exposure to contaminated surface water to an increased risk of sporadic gastroenteritis, especially in children^5^. Risk assessments suggest that noroviruses and *Cryptosporidium* spp. are major causative agents of sporadic gastroenteritis in swimmers in non-outbreak settings along with *Giardia lamblia*, *Campylobacter* spp., adenoviruses, and rotaviruses^3,4,6^. Seroconversion can be used in prospective study settings to detect incident infections caused by specific pathogens. However, the invasive nature of blood sampling makes it a problematic approach in longitudinal community-based studies, especially those involving children. Non-invasive salivary antibody assays are potentially very useful tools in epidemiological surveillance of waterborne infections if technical challenges associated with lower antibody levels and varying sample composition are resolved^7^. Salivary antibody responses to noroviruses correlate with systemic antibody levels and blocking of norovirus binding to target human antigens^8^. Salivary antibody assays for noroviruses and *Cryptosporidium* have been developed by several groups^9–13^. There are examples of successful application of these assays in epidemiological studies of drinking water-related infections^14,15^, as well as studies of recreational water-related infections involving saliva sampling by mail and detecting salivary antibody conversions (hereafter called “seroconversions”) to these pathogens^16,17^.

Noroviruses have a short incubation period which usually varies from 1 to 4 days. Typical symptoms of self-resolving norovirus gastroenteritis include vomiting, nausea, diarrhea, and stomachache. Noroviruses are the most common cause of gastroenteritis in adults in the US^18^. Two genogroups are responsible for most human infections: genogroup I (GI) noroviruses are less common but more likely to be environmentally transmitted; genogroup II (GII) noroviruses are more often transmitted through person-to-person contacts although waterborne outbreaks caused by GII noroviruses are also reported regularly^19^. There are tens of norovirus genotypes and variants; the currently dominant epidemic genotype GII.4 causes a majority of human norovirus infections^20^. Antibody responses to noroviruses mature during the first several years of life when children experience multiple infections^21^. Antibody responses to a specific norovirus variant can cross-react with other variants with a greater cross-reactivity within the same genogroup^20^. Immunoglobulin (Ig) G response to noroviruses typically occurs within 2 weeks of infection and remains elevated for several months, longer than other antibody isotypes^22^. An IgG response is a preferred marker of norovirus infection due to its longer persistence and higher sensitivity^11^.

*Cryptosporidium* is a protozoan parasite that forms infectious oocysts that are resistant to environmental degradation and chemical disinfection. Two species commonly infect humans: *C. hominis* specializes in parasitizing humans while various strains of *C. parvum* can be transmitted to humans from animals^23^. Gastroenteritis caused by this parasite can result in watery diarrhea and other gastrointestinal symptoms and is called cryptosporidiosis. Although most cases of cryptosporidiosis in the US are mild and self-limiting, this parasite can cause severe and life-threatening illness in immuno compromised individuals; it is a major contributor to the burden of gastrointestinal disease in young children in low and middle income countries^24^. Cryptosporidiosis is not as common as norovirus gastroenteritis but the true incidence is likely underreported^25,26^. It has a longer incubation period than norovirus gastroenteritis averaging approximately 1 week. An IgG response to the immunodominant 17 kDa antigen of *Cryptosporidium* usually occurs within 2 weeks and remains elevated for months^27^. Serum and salivary antibody tests utilizing a recombinant version of the antigenically identical recombinant 15 kDa sporozoite surface glycoprotein which is also called gp15^28,29^ have been applied in epidemiological studies^14,29^. Antibody responses mature in children and level off in adults^14,15,30^.

The objective of this study was to assess the risk of symptomatic and asymptomatic infections with noroviruses and *Cryptosporidium* in beachgoers at a Great Lakes beach using an in-house salivary antibody assay developed at the US Environmental Protection Agency (EPA).

## Results

The study involved recruitment of beachgoers at a public beach in Racine, WI with collection of water exposure and demographic information, and a baseline saliva sample (S1) immediately following recruitment. A follow-up phone interview to collect data on gastrointestinal and other symptoms was scheduled approximately 10 days later. Two follow-up saliva samples were self-collected by study participants and mailed to the EPA laboratory approximately 2 weeks (S2) and 30–40 days after recruitment (S3). This study was conducted before and during a separate intervention study aimed at removing gulls from the beach in an effort to reduce water pollution with pathogens that can be carried by gulls. The goal of the present study was to describe risk factors associated with waterborne infections caused by human-relevant noroviruses and *Cryptosporidium* spp., which are unlikely to be transmitted by gulls. This study does not focus on the gull removal intervention, but rather builds upon a body of work which evaluates salivary antibody responses to select waterborne pathogens in diverse populations across the United States.

A total of 1639 individuals were enrolled in this study and provided questionnaire data. Of these, 1517 (93%) individuals provided at least one saliva sample with an average of 2.43 samples per person; 872 (53%) individuals representing 395 households who provided all three saliva samples of sufficient volume (generally at least 0.1 mL), valid questionnaire data on age and recreational water exposure, and valid follow-up data on gastrointestinal symptoms were included in data analysis (Table 1). The mean age of these study participants was 25.8 years; almost half of them were children (Table 2). About 60% of 872 participants reported female sex and 89% reported themselves as being White. Follow-up phone interviews were conducted on average 10.4 days after the baseline beach visit (median interval 10 days, range from 9 to 21 days). A total of 110 (12.6%) participants reported episodes of gastrointestinal symptoms (diarrhea, vomiting, nausea, or stomachache during at least 1 day) between the baseline beach visit and phone interview with an incidence rate of 4.4 cases per person per year (Table 2). These included 57 individuals (6.5% of all participants) who reported gastrointestinal symptoms occuring during the first 4 days after the beach visit (Table 2) with an incidence rate of 6.0 cases per person per year.

**Table 1:** Enrollment and sample collection.

Set	Number of individuals	Percent of individuals (%)	Households	Percent of households (%)	Average household size	Number of samples	Average samples per person	Percent of initial samples (%)
All individuals enrolled in the study	1639	100	675	100	2.43			
Contributed at least one saliva sample	1517	93	675	100	2.25	3681	2.43	100
Included in laboratory tests	906	55	403	60	2.25	2702	2.98	73
Included in final data analysis	872	53	395	59	2.21	2616	3.00	71

**Table 2:** Descriptive statistics of the study population.

Category	Level	N or mean	Column percent or SD
All		872	100%
Age, mean and SD		25.8	19.3
Age category	Adults	446	51.1%
	Children	426	48.9%
Gender	Female	523	60.0%
	Male	339	38.9%
	Not reported	10	1.1%
Race	White	774	88.8%
	Black or African American	16	1.8%
	Asian or Native Hawaiian or Other Pacific Islander	11	1.3%
	American Indian or Alaskan Native	5	0.6%
	Other	38	4.4%
	Not reported	28	3.2%
Ethnicity	Not Hispanic	691	79.2%
	Hispanic	62	7.1%
	Not reported	119	13.6%
Episodes of symptoms within the interval between beach visit and interview (mean 10.4 days)	No symptoms	762	87.4%
	Any gastroenteritis symptoms	110	12.6%
	Vomiting	15	1.7%
	Diarrhea	59	6.8%
	Nausea	33	3.8%
	Stomachache	82	9.4%
Episodes of symptoms within the first 4 days of the beach visit	No symptoms	815	93.5%
	Any gastroenteritis symptoms	57	6.5%
	Vomiting	6	0.7%
	Diarrhea	32	3.7%
	Nausea	17	1.9%
	Stomachache	42	4.8%

**Figure 1:**
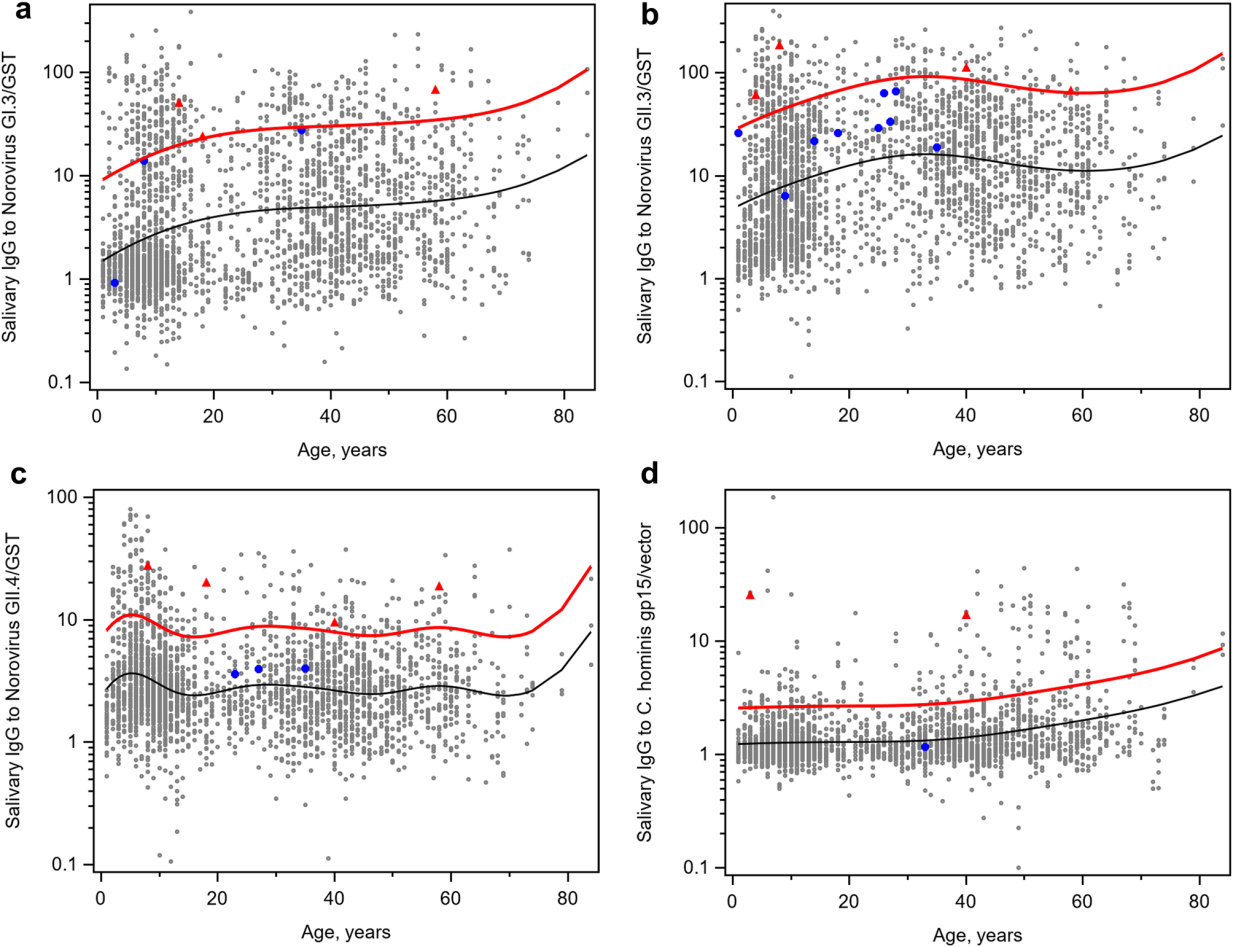
Flexible age-dependent cut-off values for seroconversion definitions based on the upper 90% prediction bound of the spline function of age (solid red line). Predicted mean is a solid black line. S2 samples corresponding to final seroconversions (strict definition) are marked with red triangles while additional S2 samples that met the preliminary seroconversion definition are marked with blue circles. (**a**) Norovirus GI.3; (**b**) Norovirus GII.3; (**c**) Norovirus GII.4 Sydney; and (**d**) *Cryptosporidium*.

A total of 17 individuals (1.9%) met the preliminary definition of seroconversion (at least a four-fold increase in response in the S2 sample relative to the S1 sample and at least three-fold increase in response in the S3 sample relative to the S1 sample) to at least one pathogen. Among these 17 people, only seven individuals (0.8% of the study population) met the final, strict definition of seroconversion with the additional requirement of a response in the S2 sample being above the upper 90% prediction bound of a corresponding spline function of age (Fig. 1). These seven individuals included three adults and four children (Table 3), all from different households. The breakdown of seroconversions by pathogen is as follows: six (0.7% of the study population) individuals seroconverted to noroviruses, including one seroconversion to GI noroviruses, three seroconversions to GII noroviruses, and two seroconversions to both norovirus genogroups during the same time interval; two persons (0.2% of the study population) had seroconversions to *Cryptosporidium* including an individual who seroconverted to *Cryptosporidium* and GII noroviruses during the same time interval. Although individuals with seroconversions (converters) were, on average, younger than non-converters (20.7 years and 25.9 years, respectively), age was not significantly associated with seroconversion status (p = 0.5).

**Table 3:** Individuals with seroconversions.

Individual	Age (years)	Crypto-sporidium	GI.3	GII.3	GII.4 Sydney	GII noroviruses	Any norovirus	Gastroenteritis symptoms	Water contact
A	14	0	1	0	0	0	1	1	Swallow
B	18	0	1	0	1	1	1	0	Swallow
C	58	0	1	1	1	1	1	0	Swallow
D	40	1	0	1	1	1	1	0	Body contact
E	8	0	0	1	1	1	1	0	Swallow
F	4	0	0	1	0	1	1	0	Swallow
G	3	1	0	0	0	0	0	0	Swallow
Mean or sum	20.7	2	3	4	4	5	6	1	NA

Study participants were distributed rather evenly among the six categories of water exposure; 176 (20%) individuals reported swallowing water (Table 4). Younger age was strongly associated with a greater degree of water exposure in a univariate test for trend (p < 0.0001). All self-reported gastrointestinal symptoms except nausea were significantly associated with ordinal water exposure categories. Seroconversions were also significantly associated with water exposure in a univariate test for trend (p = 0.003, Table 4).

**Table 4:** Recreational water exposure and gastrointestinal symptoms.

Category	N	Percent	Age		Seroconverted		Any GI symptoms		Vomiting		Diarrhea		Nausea		Stomachache	
			Mean	SD	N	Percent	N	Percent	N	Percent	N	Percent	N	Percent	N	Percent
No water contact	109	13%	39.0	15.7	0	0.0%	7	6.4%	1	0.9%	4	3.7%	3	2.8%	5	4.6%
Legs only	130	15%	37.1	18.5	0	0.0%	14	10.8%	1	0.8%	6	4.6%	4	3.1%	9	6.9%
Body contact but head not immersed	159	18%	33.5	17.3	1	0.6%	19	11.9%	1	0.6%	10	6.3%	5	3.1%	13	8.2%
Immersed head but no water in the mouth	123	14%	22.3	19.6	0	0.0%	17	13.8%	2	1.6%	9	7.3%	3	2.4%	14	11.4%
Water in the mouth but not swallowed	175	20%	19.3	16.4	0	0.0%	23	13.1%	5	2.9%	11	6.3%	9	5.1%	18	10.3%
Swallowed water	176	20%	11.4	11.3	6	3.4%	30	17.0%	5	2.8%	19	10.8%	9	5.1%	23	13.1%
Test for trend	NA		p < 0.0001		p = 0.003		p = 0.01		p = 0.05		p = 0.02		p = 0.18		p = 0.008	

Six of seven individuals who met the strict definition of seroconversion (85.7%) reported swallowing lake water while only 170 of 860 individuals who did not seroconvert (19.7%) reported swallowing water. In contrast to the pattern in the entire study population, converters tended to be older than non-converters among those who swallowed water (average 17.5 years vs. 11.1 years) as well as among those individuals who did not swallow water (40.0 years was the age of a single person who had a seroconversion vs. 29.4 years average age in non-converters). Therefore, adjusting for age was necessary when assessing the effect of water exposure on conversions in regression analysis (see below). Finally, there were four (1.1%) seroconversions before the gull removal intervention and three (0.6%) seroconversions after it; the effect of intervention on seroconversions was not significant (p = 0.4).

In a univariate logistic regression analysis, the crude odds ratio of seroconversion to norovirus or *Cryptosporidium* in individuals who swallowed water vs. all other study participants was 24.5 (2.9; 205), p = 0.003; after adjusting for age, this odds ratio increased to 49.5 (4.5; 549), p = 0.0015. The effect of age was short of being significant in this model (p = 0.17) but adjusting for age changed the estimated effect of swallowing water substantially. The crude risk difference of seroconversion associated with swallowing water was 32.7 (5.7; 59.6) per 1000 beachgoers. The age-standardized risk difference with 95% bootstrap confidence interval was 94.8 (4.6; 276) per 1000 beachgoers. This estimate corresponds to a risk difference in the situation when both exposure groups have the same age distribution as the entire study population.

Among the six individuals who seroconverted to norovirus, one person (16.7%) reported symptoms of diarrhea, nausea, and vomiting. These symptoms occurred within 4 days of the baseline beach visit, which is consistent with a short incubation period of noroviruses. The remaining 56 episodes of any gastrointestinal symptoms, including five episodes involving vomiting with or without other gastrointestinal symptoms, 16 episodes involving nausea, and 31 episodes involving diarrhea occurred among 866 individuals who did not seroconvert to noroviruses (rates 6.5%, 0.6%, 1.8%, and 3.6%, respectively). Despite the small sample size, there were significant associations between norovirus seroconversion and episodes of gastrointestinal symptoms involving vomiting with an odds ratio of 34 (3.4; 350), p = 0.003, and with episodes of gastrointestinal symptoms involving nausea with an odds ratio of 11 (1.2; 96), p = 0.04. Associations between norovirus seroconversions and episodes of any gastrointestinal symptoms as well as episodes involving diarrhea with or without other symptoms were not significant, with odds ratios of 2.9 (0.3; 25), p = 0.3, and 5.4 (0.6; 48), p = 0.1, respectively.

## Discussion

This prospective salivary antibody study demonstrated a strong and statistically significant association between self-reported water exposure among beachgoers at a Lake Michigan beach and seroconversions to noroviruses or *Cryptosporidium* spp. In addition, there was a significant association between episodes of vomiting or nausea within 4 days of the beach visit and seroconversion to noroviruses. This is the first salivary antibody study in beachgoers that demonstrated both associations—between water exposure and seroconversion, and between seroconversion and gastrointestinal symptoms—in the same set of participants. Previous studies in beachgoers using similar methodology demonstrated either a statistically significant association between water exposure and asymptomatic seroconversions to a norovirus^16^, or between norovirus seroconversions and gastrointestinal symptoms^17^. Other previous salivary antibody studies demonstrated significant associations between gastrointestinal symptoms and seroconversions to noroviruses^14^ and *Cryptosporidium*^14,15^. One of these studies also demonstrated a significant association between using swimming pools and seroconversions to *Cryptosporidium*^14^. Unlike EPA’s previous salivary antibody studies in beachgoers, the present study included testing for salivary IgG responses to *Cryptosporidium*. However, the number of detected seroconversions was too small to analyze *Cryptosporidium* data separately. Therefore, statistical tests of associations utilized data on all seroconversions or norovirus seroconversions only.

The present study used three norovirus recombinant proteins representing GI.3, GII.3 and GII.4 Sydney variants. EPA’s previous salivary antibody studies used different sets of norovirus proteins produced using the same method and acquired from the same source, Cincinnati Children’s Hospital Medical Center. For example, a study in Massachusetts used proteins of GI.1, GII.4 VA387, and GII.9 noroviruses^14^, a study in beachgoers in Puerto Rico used proteins of GI.1 and GII.4 VA387 noroviruses^16^, and a study in beachgoers at a different Lake Michigan beach used proteins of GI.1, GI.3, GII.3, GII.4 VA387, and GII.9 noroviruses^17^. The latter study included a very similar subset of proteins, the same saliva sampling design, and the definition of seroconversion as the present study. That study found an overall 2.1% seroconversion rate. If seroconversions to GI.1 and GII.9 noroviruses were excluded to make the sets of proteins in that study almost identical to the present study (except using protein of a different GII.4 norovirus variant), the seroconversion rate would be 1.0%, which is similar to the 0.7% norovirus seroconversion rate in the present study. The rate of self-reported swallowing of water in the previous Lake Michigan study (35.7%) was higher than in the present study; the association of norovirus seroconversions with swallowing water was positive but short of being significant. The differences in study results could be due to a random effect as both studies had small numbers of seroconversions.

Antibodies to specific noroviruses can cross-react to heterologous noroviruses, especially those in the same genogroup^20^. Infections with noroviruses which are not included in the multiplex assay, however, are likely to produce a weaker observed antibody response potentially affecting the assay’s sensitivity. The above comparison of results from two similar studies shows that an assay with a broader set of norovirus proteins is likely to capture more norovirus infections. At the same time, including more recombinant proteins increases the cost of analysis. An optimal assay would involve a parsimonious set of norovirus proteins that optimizes the trade-off between the cost of analysis and sensitivity of the seroconversion test. The composition of such assay would need to be periodically adjusted to take in account the rapidly evolving noroviruses circulating in the target population. The present study likely missed some norovirus infections. Therefore, it might underestimate the absolute risk of norovirus infections attributable to ingesting lake water. In addition, this study did not involve testing saliva for antibodies specific to many other pathogens that can be transmitted via recreational water as well as various other pathways, such as adenoviruses, rotaviruses, *Campylobacter* spp., and *Giardia lamblia*.

The rate of episodes of gastrointestinal symptoms in this study (4.4 cases per person-year on average, 6.0 cases per person-year during the first 4 days after the beach visit, 3.5 cases per person-year during the rest of follow-up) was higher than the rate of gastroenteritis in many previously conducted studies, which varied from 0.1 to 3.5 cases per person-year^31^. This difference could be in part due to stricter definitions of gastroenteritis used in previous studies (e.g., at least three episodes of diarrhea during a day, or symptoms lasting more than 1 day) as opposed to any episodes of symptoms. In the present study, the research hypothesis was known to the participants who might consciously or unconsciously overreport symptoms during the first 4 days after the beach visit. Some episodes of gastrointestinal symptoms immediately after the beach visit could also be caused by food poisoning during the beach visit or other weekend activities. The rate of reported symptoms during the rest of follow-up was within the range of previously reported data.

Only one report of gastrointestinal symptoms during the first 4 days after the beach visit was linked to a norovirus seroconversion. The proportion of episodes of gastrointestinal symptoms attributable to noroviruses was 1.8%. The Wilson’s 95% confidence interval for this proportion is very wide ranging from 0.3 to 9.3%. Noroviruses are a main cause of gastroenteritis accounting for about 13% of gastroenteritis-associated ambulatory health care visits in the US^32^. However, norovirus infections typically peak in winter in the US while the contribution of noroviruses to outpatient gastroenteritis cases in August can be as low as approximately 2%^18^.

In this study, 17 individuals experienced seroconversions to noroviruses or *Cryptosporidium* under a preliminary, broad definition of seroconversion (see the “Methods” section); of them, only seven individuals were classified as seroconverters using the final, strict seroconversion definition incorporating a flexible age-dependent cut-off as an additional criterion. Using the preliminary seroconversion definition, an age-adjusted odds ratio of seroconversion was 5.0 (1.6; 15.5) among those who swallowed water compared to all other participants. This odds ratio estimate is almost ten times smaller than the corresponding estimate produced using the final, strict definition of seroconversion, 49.5 (4.5; 549). At the same time, the age standardized risk differences between those who swallowed water and those who did not swallow water under the preliminary, broad seroconversion definition, 105 cases per 1000 beachgoers (not shown in the Results section), was very similar to the risk difference estimate under the final, strict seroconversion definition, 95 cases per 1000 beachgoers. The marked increase in odds ratio estimate in the model using the final, strict seroconversion definition was because seroconversions in the unexposed group decreased much more than in the exposed group after applying the additional seroconversion criterion to meet the final definition: the reductions were from eight to one seroconversion in the unexposed group, and from nine to six seroconversions in the exposed group. The final, strict definition was likely to produce a greater specificity and a lower sensitivity of the seroconversion test. As was discussed previously^14^, a study of rare outcomes, such as sporadic norovirus or *Cryptosporidium* infections, should maximize specificity, even at the cost of sacrificing some sensitivity, in order to increase the proportion of true positive results. This approach reduces the bias towards the null effect in regression analysis due to non-differential outcome misclassification.

A previously conducted salivary antibody study in beachgoers demonstrated that recreational exposure to sewage-contaminated water was associated with an increased risk of norovirus infections^16^; a similar study in beachgoers at a different Lake Michigan beach produced a positive but statistically non-significant association between recreational water exposure and norovirus seroconversion^17^. The objective of the present study was to provide further information on waterborne infection risks using a similar norovirus antibody assay, as well as a *Cryptosporidium* antibody assay which was successfully used in a previous study of drinking water-related infections^14^. The main sources of these pathogens at the North Beach in Racine, WI were unknown; they may have included urban stormwater outfalls, non-point sources of human fecal pollution, such as surface runoff^33^, or possible shedding from the swimmers themselves. Oocysts of *C. parvum*, of the two main species of *Cryptosporidium* infecting humans, could also be excreted by non-human mammals, including wildlife and farm animals.

This study was conducted before and during an intervention to remove gulls from the beach. The intervention aimed to reduce water pollution with other pathogens that can be carried by gulls. It produced a dramatic reduction in the levels of bacterial indicators of fecal pollution in the water^34^. Feces of gulls at the North Beach in Racine, WI were shown to contain human pathogens: *Campylobacter* spp. was by far the most common followed by *Salmonella* spp.^35^; however, gulls are unlikely to carry human noroviruses. While some *Cryptosporidium* species, such as *C. baileyi*, *C galli*, and *C. meleagridis*, are known to infect birds, these species do not cause infections in humans, with a possible exception of *C. meleagridis*^36,37^. Therefore, it is not surprising that the present analysis did not produce evidence of a reduction in norovirus and *Cryptosporidium* infections during the intervention. Moreover, statistical power was quite limited to test the effects of an intervention on specific incident infections. It is possible that the intervention reduced incidence rates of waterborne infections with *Campylobacter* spp. and other pathogens that are carried by gulls; these potential effects were not investigated.

The main limitation of the present study, as well as EPA’s previous two salivary antibody studies in beachgoers^16,17^, is the small sample size and the small number of detected seroconversions. However, these three studies together provide strong evidence of sporadic transmission of symptomatic and asymptomatic norovirus infections via recreational water exposure and demonstrate the utility of a non-invasive salivary immunoassay in population-based observational studies.

## Methods

### Study design

The study design involved collection of water exposure and demographic information from beachgoers at a public beach, a follow-up phone interview to collect data on gastrointestinal and other symptoms 10–12 days later, and collection of three saliva samples at baseline (S1) and two follow-up sampling events with target intervals from baseline of approximately 2 weeks (S2) and 30–40 days (S3). This sampling design is consistent with two previous EPA salivary antibody studies^16,17^ and an earlier epidemiological study among beachgoers^38^.

### Study site

This study was conducted at the popular North Beach on Lake Michigan at the mouth of the Root River in Racine, Wisconsin in August 2011 alongside a water quality monitoring survey. The later part of the study was conducted concurrently with efforts to remove gulls from the beach, which produced substantial improvements in the levels of fecal indicator bacteria, *E. coli* and *Enterococci*^34^. The goal of the salivary antibody study was to describe risk factors associated with two common waterborne infections, human noroviruses and *Cryptosporidium* spp. This study was not focused on the impacts of the gull removal but was conducted as part of the research team’s efforts to apply previously developed in-house salivary antibody tests in diverse populations across the United States.

### Study population

This observational study involving human participants was approved by the Institutional Review Board at the University of North Carolina at Chapel Hill under an agreement with EPA, and by an EPA human subjects research review official. All methods were performed in accordance with the relevant guidelines and regulations. The target population was individuals and families with at least one adult capable of communicating in English or Spanish. Beachgoers were recruited at the beach on weekends. Adults 18 years and older provided signed informed consent for themselves and for their children under 7 years of age. Children aged 7–14 years and adolescents aged 15–17 years signed different informed assent forms, along with signed parental consent. A baseline sociodemographic and exposure questionnaire was administered at the beach. Recreational water exposure was characterized using six ordinal mutually exclusive categories: (1) no contact with water; (2) legs only contact; (3) full body contact but head not submerged; (4) head submerged but no water in the mouth; (5) water in the mouth but not swallowed; and (6) water swallowed.

Data on gastrointestinal symptoms including diarrhea, vomiting, nausea, and stomachache, and dates of symptom onset were collected by phone approximately 10–12 days later from a contact adult who answered on behalf of all household members participating in the study. All episodes of gastrointestinal symptoms of any duration were included in statistical analysis. Each participant could have only one episode of symptoms between the beach visit and follow-up interview.

### Saliva sampling

Samples were collected using a sponge-type Oracol oral fluid sampling device (Malvern Medical Developments, Worcester, United Kingdom) designed to collect saliva enriched with gingival crevicular fluid containing serum IgG^39^. The Oracol sampler consists of a 15 mL sampling tube and a cylindrical sponge with a handle. Sampling involves rubbing the gums to squeeze out the gingival fluid for one minute or until the sponge becomes fully saturated.

Baseline S1 samples were collected by study participants in the presence of survey personnel at the beach after a demonstration of sampling technique. Samples were immediately stored on ice, then frozen in the field office, and shipped to the EPA laboratory in insulated containers with ice packs. Follow-up S2 and S3 samples were self-collected by study participants at home. Sampling kits were mailed to participating households in insulated containers with sample labels, ice packs, and pre-paid return overnight shipping labels. Participants were instructed to freeze ice packs and saliva samples before shipping them to EPA. When needed, up to two reminder phone calls were made to prompt study participants to return their samples. Upon delivery to the laboratory, saliva samples were processed to separate saliva from the sampling sponge and debris by centrifugation and stored in 1.5 mL microcentrifuge tubes at − 80 °C until analysis. Study participants received $ 5 for the initial saliva sample, and $ 20 for each additional saliva sample, for a maximum of $ 45.

### Laboratory analysis

Samples were analyzed for immunoglobulin (Ig) G responses to three noroviruses, including a common genogroup I (GI) norovirus GI.3, and two common GII noroviruses, GII.3 and GII.4 Sydney variant, and *Cryptosporidium* using an in-house multiplexed suspension immunoassay on the Luminex platform (Luminex Corp. Austin, TX) which was developed previously^10,11^.

The recombinant protruding (P) domains of norovirus capsid proteins were procured from Dr. Xi Jiang’s laboratory at Cincinnati Children's Hospital Medical Center (Cincinnati, OH). These antigens were produced using an *E. coli* expression system with a GST purification tag as described previously^40^. The recombinant gp15 (15 kDa) protein of *Cryptosporidium hominis* TU502 isolate was cloned and purified as described previously^29^. The gp15 protein is an immunogenic zoite surface antigen. As antibody responses to gp15 antigens from *C. hominis* and *C. parvum* are strongly correlated^29^, a seroconversion to the gp15 antigen of *C. hominis* can capture infections with both *Cryptosporidium* species. In addition, salivary IgG responses to norovirus recombinant protein purification tag glutathione-S-transferase (GST), and *Cryptosporidium* control protein containing the thioredoxin tag, S-tag, and His-tag^29^ hereafter called “empty vector”, were measured as internal assay controls for a total of six proteins in the assay.

The proteins included in the assay were covalently coupled to spectrally distinct sets of MagPlex Luminex microspheres using the standard Luminex carbodiimide protein coupling protocol described in the Luminex xMAP® cookbook (https://info.luminexcorp.com/en-us/research/download-the-xmap-cookbook, accessed on July 13, 2021). The 50 mM 2-(*N*-morpholino) ethanesulfonic acid (MES) buffer, pH 5.0 was used in all coupling reactions. Proteins were added to the corresponding 500 µL coupling reaction solutions at the following optimized amounts: *C. hominis* gp15 and norovirus GII.4 Sydney—25 µg each; GST—20 µg; GI.3, GII.3, and empty vector—10 µg each. Coupling confirmation tests were conducted using serially diluted guinea pig anti-norovirus antibody from Cincinnati Children’s Hospital, rabbit anti-*Cryptosporidium* gp15 from Tufts Medical Center, rabbit anti-GST antibody (Invitrogen, Carlsbad, CA, cat. A-5800), and serially diluted control serum samples, with corresponding secondary anti-species antibodies as described previously^10,14^.

Saliva samples were diluted in the standard Luminex assay buffer 1:2 prior to analysis for a final 1:4 dilution in the microplate well. Sample sets which included a saturated response to at least one protein were re-tested at 1:8 preliminary dilution. Samples were analyzed using the standard Luminex assay protocol. Signal was detected using a biotinylated donkey anti-human IgG Fc-specific antibody (Jackson ImmunoResearch Inc., West Grove, PA) and streptavidin R-phycoerythrin conjugate (SAPE; Invitrogen, Carlsbad, CA). Plates were read using a MAGPIX Luminex plate reader. Results were expressed as Median Fluorescent Intensity (MFI) values calculated from at least 50 microspheres of each type. Each plate included at least three blank wells and three control serum mixes, which were prepared by mixing selected serum samples acquired from a commercial biobank and leftover serum samples from a previously conducted US EPA study. Each control mix was assayed at three serial dilutions.

All samples from the same individual were assayed on one plate in order to minimize assay variability. Approximately 15% of sample sets, including most sample sets with initial results suggesting a seroconversion, were re-tested on a different plate.

### Statistical data analysis

Data were analyzed using SAS version 9.4 (SAS Institute, Cary, NC, USA). To control for potential sample-to-sample variability in saliva composition, all antibody responses were defined as ratios of MFI values for the target antigen to its purification tag. Specifically, responses to noroviruses were defined as a ratio of anti-norovirus IgG to anti-GST IgG, and responses to *Cryptosporidium* were defined as a ratio of anti-*Cryptosporidium* IgG to anti-empty vector IgG.

Only individuals who returned all three usable saliva samples and necessary data (e.g. provided sufficient sample volume and valid questionnaire and symptom data) were included in data analysis. A preliminary definition of seroconversion as a marker of incident infection included at least a four-fold increase in response in the S2 sample relative to the S1 sample and at least three-fold increase in response in the S3 sample relative to the S1 sample. In order to remove potentially false-negative results, increase the specificity of seroconversion tests, and account for the association between antibody responses and age, the final definition also included a requirement that an antibody response in the S2 sample had to be above the upper bound of a one-sided 90% predictive confidence interval from a spline function of pathogen-specific antibody responses on age. Penalized B-Spline functions were fitted to log-transformed antibody response data using SAS procedure ***transreg***. Curve parameters were selected automatically by minimizing the Schwartz’s Bayesian Information Criterion. When saliva volume allowed retesting all samples, only those seroconversions that were confirmed at re-testing were counted in further analysis.

Associations between recreational water exposure and seroconversions were tested using a Cochran-Armitage Trend Test (procedure ***freq***). A univariate association between water exposure and age was assessed using Somers’ D test (procedure ***freq***). Odds ratios of seroconversion in exposed vs. unexposed, and odds ratios of gastrointestinal symptoms in converters vs. non-converters with 95% confidence intervals were estimated using logistic regression models (procedure ***logistic***) with adjusting for age. Age-standardized risk differences of seroconversion in exposed and unexposed individuals were estimated using marginal structural binomial models and robust 95% confidence intervals were estimated by bootstrapping as described previously^41^. This method is a flexible extension of a direct standardization method. A weight is assigned to each person which is equal to an inverse probability of exposure conditional on confounders (in this case, on age). Predicted probability of exposure is estimated using logistic regression of a binary exposure variable on confounders. Marginal probability of exposure is estimated at next stage by fitting a logistic regression model with no covariates. Weights are calculated using predicted and marginal probabilities. Finally, a linear binomial regression model is used to calculate adjusted risk difference, and a bootstrap method is applied to estimate a percentile-based confidence interval.

### Disclaimer

The views expressed in this manuscript are those of the authors and do not represent EPA policy. Mention of trade names does not imply endorsement.

## Data Availability

This dataset contains personally identifiable information (PII) collected with institutional review board review and approval, and an informed consent process which indicated individuals would not be identified as a result of their participation.

## References

[1] Graciaa DS, Cope JR, Roberts VA, Cikesh BL, Kahler AM, Vigar M (2018). Outbreaks associated with untreated recreational water—United States, 2000–2014. MMWR Morb. Mortal. Wkly. Rep..

[2] Hlavsa MC, Roberts VA, Kahler AM, Hilborn ED, Mecher TR, Beach MJ (2015). Outbreaks of illness associated with recreational water-United States, 2011–2012. MMWR Morb. Mortal. Wkly. Rep..

[3] Boehm AB, Graham KE, Jennings WC (2018). Can we swim yet? Systematic review, meta-analysis, and risk assessment of aging sewage in surface waters. Environ. Sci. Technol..

[4] Soller JA, Bartrand T, Ashbolt NJ, Ravenscroft J, Wade TJ (2010). Estimating the primary etiologic agents in recreational freshwaters impacted by human sources of faecal contamination. Water Res..

[5] Arnold BF, Wade TJ, Benjamin-Chung J, Schiff KC, Griffith JF, Dufour AP (2016). Acute gastroenteritis and recreational water: highest burden among young US children. Am. J. Public Health.

[6] Purnell S, Halliday A, Newman F, Sinclair C, Ebdon J (2020). Pathogen infection risk to recreational water users, associated with surface waters impacted by de facto and indirect potable reuse activities. Sci. Total Environ..

[7] Exum NG, Pisanic N, Granger DA, Schwab KJ, Detrick B, Kosek M (2016). Use of pathogen-specific antibody biomarkers to estimate waterborne infections in population-based settings. Curr. Environ. Health Rep..

[8] Tamminen K, Malm M, Vesikari T, Blazevic V (2018). Norovirus-specific mucosal antibodies correlate to systemic antibodies and block norovirus virus-like particles binding to histo-blood group antigens. Clin. Immunol..

[9] Moe CL, Sair A, Lindesmith L, Estes MK, Jaykus LA (2004). Diagnosis of norwalk virus infection by indirect enzyme immunoassay detection of salivary antibodies to recombinant Norwalk virus antigen. Clin. Diagn. Lab. Immunol..

[10] Griffin SM, Chen IM, Fout GS, Wade TJ, Egorov AI (2011). Development of a multiplex microsphere immunoassay for the quantitation of salivary antibody responses to selected waterborne pathogens. J. Immunol. Methods.

[11] Griffin SM, Converse RR, Leon JS, Wade TJ, Jiang X, Moe CL (2015). Application of salivary antibody immunoassays for the detection of incident infections with Norwalk virus in a group of volunteers. J. Immunol. Methods.

[12] Pisanic N, Ballard SB, Colquechagua FD, Francois R, Exum N, Yori PP (2019). Minimally invasive saliva testing to monitor norovirus infection in community settings. J. Infect. Dis..

[13] Moss DM, Montgomery JM, Newland SV, Priest JW, Lammie PJ (2004). Detection of cryptosporidium antibodies in sera and oral fluids using multiplex bead assay. J. Parasitol..

[14] Egorov AI, Griffin SM, Ward HD, Reilly K, Fout GS, Wade TJ (2018). Application of a salivary immunoassay in a prospective community study of waterborne infections. Water Res..

[15] Egorov AI, Montuori Trimble LM, Ascolillo L, Ward HD, Levy DA, Morris RD (2010). Recent diarrhea is associated with elevated salivary IgG responses to Cryptosporidium in residents of an eastern Massachusetts community. Infection.

[16] Wade TJ, Augustine SAJ, Griffin SM, Sams EA, Oshima KH, Egorov AI (2018). Asymptomatic norovirus infection associated with swimming at a tropical beach: A prospective cohort study. PLoS ONE.

[17] Wade TJ, Griffin SM, Egorov AI, Sams E, Hudgens E, Augustine S (2019). Application of a multiplex salivary immunoassay to detect sporadic incident norovirus infections. Sci. Rep..

[18] Grytdal SP, DeBess E, Lee LE, Blythe D, Ryan P, Biggs C (2016). Incidence of norovirus and other viral pathogens that cause acute gastroenteritis (AGE) among Kaiser Permanente Member Populations in the United States, 2012–2013. PLoS ONE.

[19] Bitler EJ, Matthews JE, Dickey BW, Eisenberg JN, Leon JS (2013). Norovirus outbreaks: A systematic review of commonly implicated transmission routes and vehicles. Epidemiol. Infect..

[20] Parra GI, Squires RB, Karangwa CK, Johnson JA, Lepore CJ, Sosnovtsev SV (2017). Static and evolving norovirus genotypes: Implications for epidemiology and immunity. PLoS Pathog..

[21] Blazevic V, Malm M, Honkanen H, Knip M, Hyoty H, Vesikari T (2016). Development and maturation of norovirus antibodies in childhood. Microbes Infect..

[22] Tacket CO, Sztein MB, Losonsky GA, Wasserman SS, Estes MK (2003). Humoral, mucosal, and cellular immune responses to oral Norwalk virus-like particles in volunteers. Clin. Immunol..

[23] Leoni F, Amar C, Nichols G, Pedraza-Diaz S, McLauchlin J (2006). Genetic analysis of cryptosporidium from 2414 humans with diarrhoea in England between 1985 and 2000. J. Med. Microbiol..

[24] Fayer R, Morgan U, Upton SJ (2000). Epidemiology of Cryptosporidium: Transmission, detection and identification. Int. J. Parasitol..

[25] Painter JE, Gargano JW, Yoder JS, Collier SA, Hlavsa MC (2016). Evolving epidemiology of reported cryptosporidiosis cases in the United States, 1995–2012. Epidemiol. Infect..

[26] Painter JE, Hlavsa MC, Collier SA, Xiao L, Yoder JS, Centers for Disease C (2015). Cryptosporidiosis surveillance—United States, 2011–2012. MMWR Suppl..

[27] Priest JW, Li A, Khan M, Arrowood MJ, Lammie PJ, Ong CS (2001). Enzyme immunoassay detection of antigen-specific immunoglobulin g antibodies in longitudinal serum samples from patients with cryptosporidiosis. Clin. Diagn. Lab. Immunol..

[28] Cevallos AM, Zhang X, Waldor MK, Jaison S, Zhou X, Tzipori S (2000). Molecular cloning and expression of a gene encoding Cryptosporidium parvum glycoproteins gp40 and gp15. Infect. Immun..

[29] Allison GM, Rogers KA, Borad A, Ahmed S, Karim MM, Kane AV (2011). Antibody responses to the immunodominant Cryptosporidium gp15 antigen and gp15 polymorphisms in a case-control study of cryptosporidiosis in children in Bangladesh. Am. J. Trop. Med. Hyg..

[30] Arnold BF (2019). Enteropathogen antibody dynamics and force of infection among children in low-resource settings. elife.

[31] Roy SL, Scallan E, Beach MJ (2006). The rate of acute gastrointestinal illness in developed countries. J. Water Health.

[32] Gastanaduy PA, Hall AJ, Curns AT, Parashar UD, Lopman BA (2013). Burden of norovirus gastroenteritis in the ambulatory setting–United States, 2001–2009. J. Infect. Dis..

[33] Kinzelman J, Byappanahalli MN, Nevers MB, Shively D, Kurdas S, Nakatsu C (2020). Utilization of multiple microbial tools to evaluate efficacy of restoration strategies to improve recreational water quality at a Lake Michigan Beach (Racine, WI). J. Microbiol. Methods..

[34] Converse RR, Kinzelman JL, Sams EA, Hudgens E, Dufour AP, Ryu H (2012). Dramatic improvements in beach water quality following gull removal. Environ. Sci. Technol..

[35] Kinzelman J, McLellan SL, Amick A, Preedit J, Scopel CO, Olapade O (2008). Identification of human enteric pathogens in gull feces at Southwestern Lake Michigan bathing beaches. Can. J. Microbiol..

[36] Nakamura AA, Meireles MV (2015). Cryptosporidium infections in birds—A review. Rev. Bras. Parasitol. Vet..

[37] Chappell CL, Okhuysen PC, Langer-Curry RC, Akiyoshi DE, Widmer G, Tzipori S (2011). Cryptosporidium meleagridis: Infectivity in healthy adult volunteers. Am. J. Trop. Med. Hyg..

[38] Wade TJ, Calderon RL, Brenner KP, Sams E, Beach M, Haugland R (2008). High sensitivity of children to swimming-associated gastrointestinal illness: Results using a rapid assay of recreational water quality. Epidemiology.

[39] Gammie A, Morris R, Wyn-Jones AP (2002). Antibodies in crevicular fluid: An epidemiological tool for investigation of waterborne disease. Epidemiol. Infect..

[40] Tan M, Fang P, Chachiyo T, Xia M, Huang P, Fang Z (2008). Noroviral P particle: Structure, function and applications in virus-host interaction. Virology.

[41] Richardson DB, Kinlaw AC, MacLehose RF, Cole SR (2015). Standardized binomial models for risk or prevalence ratios and differences. Int. J. Epidemiol..

